# [μ-(4*S*,5*S*,15*S*,16*S*)-10,21-Di-*tert*-butyl-4,5,15,16-tetra­phenyl-3,6,14,17-tetra­aza­tricyclo­[17.3.1.1^8,12^]tetra­cosa-1(23),8,10,12(24),19,21-hexa­ene-23,24-diolato-κ^8^ *N* ^3^,*N* ^6^,*O* ^23^,*O* ^24^:*N* ^14^ *N* ^17^,*O* ^23^,*O* ^24^]bis­[(acetato-κ*O*)zinc(II)] ethanol disolvate

**DOI:** 10.1107/S1600536809021990

**Published:** 2009-06-17

**Authors:** Li-Jing Fan, Jian-Fang Ma, Jie Liu

**Affiliations:** a Department of Chemistry, Northeast Normal University, Changchun 130024, People’s Republic of China

## Abstract

In the title compound, [Zn_2_(C_36_H_42_N_4_O_2_)(CH_3_COO)_2_]·2CH_3_CH_2_OH, a centrosymmetric dinuclear zinc macrocyclic complex is accompanied by two half-occupied ethanol solvent molecues resulting in a 1:2 macrocycle–solvent composition. The Zn^II^ atom has a square-pyramidal geometry arising from an N_2_O_3_ donor set, being coordinated by two N atoms and two O atoms from the macrocyclic ligand in the equatorial sites and one O atom from an acetate anion in the apical site. The two Zn^II^ atoms are linked by two phenolate O atoms, generating a four-membered Zn_2_O_2_ ring at the centre of the macrocycle. The *tert*-butyl group shows rotational disorder over two sets of sites in a 0.552 (12):0.448 (12) ratio. In the crystal, N—H⋯O and O—H⋯O hydrogen bonds are seen and a short intra­molecular C—H⋯O contact occurs.

## Related literature

For background to the biochemistry of zinc compounds, see: Lipscomb & Straeter (1996 ▶); Burley *et al.* (1990 ▶); Roderick & Mathews (1993 ▶); Bazzicalupi *et al.* (1997 ▶). For related structures, see: Dutta *et al.* (2005 ▶); Liu *et al.* (2007 ▶). For further synthetic details, see: Tian *et al.* (1999 ▶).
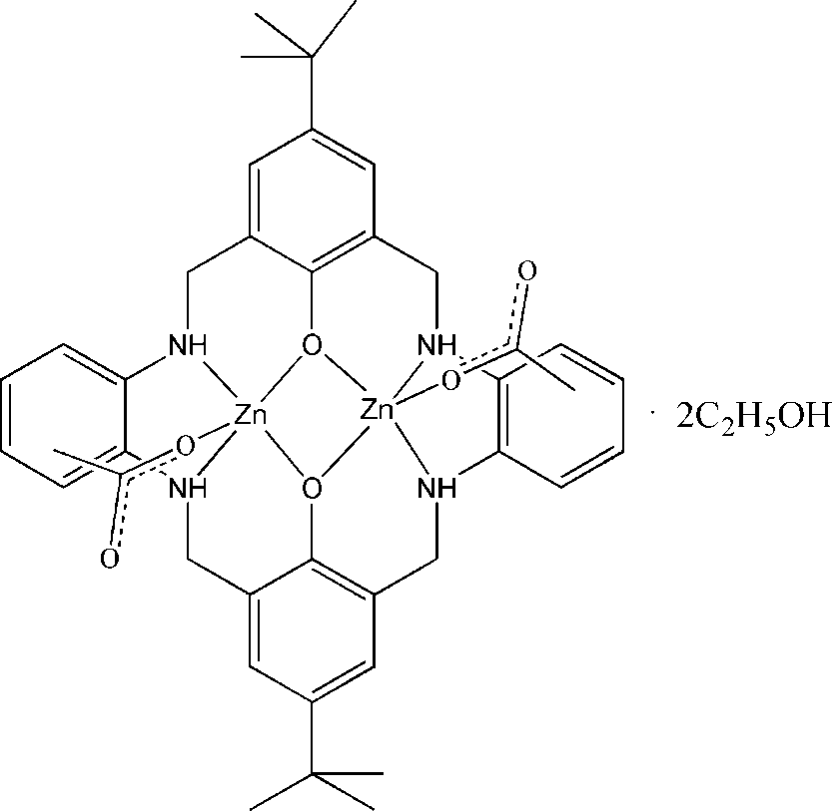


## Experimental

### 

#### Crystal data

[Zn_2_(C_36_H_42_N_4_O_2_)(C_2_H_3_O_2_)_2_]·2C_2_H_6_O
*M* *_r_* = 903.70
Triclinic, 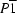

*a* = 9.0566 (3) Å
*b* = 10.8410 (5) Å
*c* = 14.2828 (5) Å
α = 71.246 (4)°
β = 86.514 (3)°
γ = 78.362 (3)°
*V* = 1300.56 (9) Å^3^
*Z* = 1
Mo *K*α radiation
μ = 0.97 mm^−1^
*T* = 293 K
0.45 × 0.25 × 0.20 mm

#### Data collection

Oxford Diffraction Gemini R Ultra diffractometer
Absorption correction: multi-scan (*CrysAlis RED*; Oxford Diffraction, 2006 ▶) *T*_min_ = 0.748, *T*_max_ = 0.824
11846 measured reflections
6368 independent reflections
4133 reflections with *I* > 2σ(*I*)
*R*_int_ = 0.035

#### Refinement

*R*[*F* ^2^ > 2σ(*F* ^2^)] = 0.077
*wR*(*F* ^2^) = 0.253
*S* = 1.05
6368 reflections
285 parameters
655 restraints
H atoms treated by a mixture of independent and constrained refinement
Δρ_max_ = 1.15 e Å^−3^
Δρ_min_ = −0.77 e Å^−3^

### 

Data collection: *CrysAlis CCD* (Oxford Diffraction, 2006 ▶); cell refinement: *CrysAlis RED* (Oxford Diffraction, 2006 ▶); data reduction: *CrysAlis RED*; program(s) used to solve structure: *SHELXS97* (Sheldrick, 2008 ▶); program(s) used to refine structure: *SHELXL97* (Sheldrick, 2008 ▶); molecular graphics: *SHELXTL* (Sheldrick, 2008 ▶); software used to prepare material for publication: *SHELXL97*.

## Supplementary Material

Crystal structure: contains datablocks global, I. DOI: 10.1107/S1600536809021990/hb2961sup1.cif

Structure factors: contains datablocks I. DOI: 10.1107/S1600536809021990/hb2961Isup2.hkl

Additional supplementary materials:  crystallographic information; 3D view; checkCIF report

## Figures and Tables

**Table 1:** Selected bond lengths (Å)

Zn1—O1	2.025 (5)
Zn1—O3	2.033 (4)
Zn1—O3^i^	2.043 (4)
Zn1—N2	2.100 (5)
Zn1—N1	2.104 (5)

Symmetry code: (i) 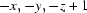.

**Table 2:** Hydrogen-bond geometry (Å, °)

*D*—H⋯*A*	*D*—H	H⋯*A*	*D*⋯*A*	*D*—H⋯*A*
C4—H4*A*⋯O2	0.97	2.45	3.246 (10)	139
N1—H1*N*⋯O4	0.86 (7)	2.23 (7)	2.952 (9)	141 (6)
N2—H2*N*⋯O5^ii^	0.87 (4)	2.13 (4)	2.999 (9)	175 (4)
O4—H4⋯O2^iii^	0.82	2.06	2.768 (10)	145
O5—H5⋯O1^iv^	0.82	1.92	2.700 (9)	159

Symmetry codes: (ii) 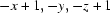; (iii) 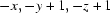; (iv) 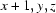.

**Fig. 1.**
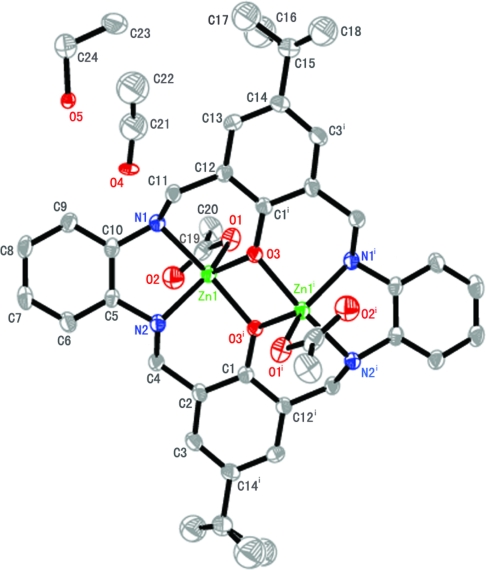
The molecular structure of (I). Displaceement ellipsoids are drawn at the 30% probability level and H atoms have been omitted for clarity.

## supplementary crystallographic information

### Comment

Zinc is an essential element for all forms of life and plays a critical role in various functions, both structural and catalytic, in proteins and enzymes (Lipscomb *et al.*,1996; Burley *et al.*,1990; Roderick & Mathews, 1993). In addition, some synthetic dinuclear zinc(II) compounds appears to have functions in dephosphorylation (Bazzicalupi *et al.*,1997). As part of out studies in this area, the title compound, (I), a new dinuclear zinc(II) compound has been synthesized, and its structure is reported here (Fig. 1).

The complete macrocycle is generated by a crystallographic inversion centre The coordination environment around zinc is a square-pyramid with two N atoms and two O atoms from the macrocyclic (C_36_H_44_N_4_O_2_) ligand occupying the basal positions and one O atom from a acetate anion occupying the apical position. The two zinc atoms are bridged by two phenolate O atoms to generate a four-membered Zn_2_O_2 _ring. The Zn—O and Zn—N distances are normal (Dutta *et al.*, 2005).

### Experimental

To a stirred methanol (30 ml) suspension of the schiff base C_36_H_40_N_4_O_2_ (0.5 mmol), which was synthesized by the methods reported previously (Tian *et al.*, 1999), was added solid NaBH_4_ (0.5 g, 13 mmol) in small portions. Over a period of 0.5 h when the red solid material gradually went into solution, and eventually an amorphous yellow mass precipitated. After 1 h, the formed yellow powder products (H_2_L) were filtrated off and washed thoroughly with water and ethanol, and dried in avacuum (yield 54%).

The title compound was prepared by reaction between the ligand (H_2_L) and zinc acetate. A mixture of H_2_L (0.108 g, 0.2 mmol) and Zn(OAc)_2_.6H_2_O (0.117 g, 0.4 mmol) in ethanol (20 ml) was heated with stirring to yield a clear pale yellow solution. Filtration and cooling to room temperature resulted in formation of a crystalline precipitate. Recrystallization by slow evaporation of an ethanol solution of the compound resulted in well-formed yellow blocks of (I) (yield 46%).

### Refinement

The N-bonded H atoms were located in a difference map and their positions were freely refined. The other H atoms were placed in calculated positions (O—H = 0.82Å, C—H = 0.93–0.96Å) and refined as riding with *U*_iso_(H) =1.2U_eq_(C,N) or 1.5U_eq_(methyl C). The methyl entities of the *tert*-butyl group are disordered over two sets of sites in a 0.552 (12):0.448 (12) ratio. The highest difference peak is 1.55 Å from O5 and the deepest difference hole is 0.77 Å from C17'.  The anisotropic displacement factors of the disordered atoms were restrained to be nearly isotropic. Additionally, the solvent of ethanol molecule is disordered in two positions (the occupancies were fixed as 0.5:0.5).

### Crystal data

**Table:**

[Zn_2_(C_36_H_42_N_4_O_2_)(C_2_H_3_O_2_)_2_]·2C_2_H_6_O	*Z* = 1
*M**_r_* = 903.70	*F*(000) = 476
Triclinic, *P*1	*D*_x_ = 1.154 Mg m^−^^3^
Hall symbol: -P 1	Mo *K*α radiation, λ = 0.71073 Å
*a* = 9.0566 (3) Å	Cell parameters from 2131 reflections
*b* = 10.8410 (5) Å	θ = 3.1–26.5°
*c* = 14.2828 (5) Å	µ = 0.97 mm^−^^1^
α = 71.246 (4)°	*T* = 293 K
β = 86.514 (3)°	Block, yellow
γ = 78.362 (3)°	0.45 × 0.25 × 0.20 mm
*V* = 1300.56 (9) Å^3^	

### Data collection

**Table:**

Oxford Diffraction Gemini R Ultra diffractometer	6368 independent reflections
Radiation source: fine-focus sealed tube	4133 reflections with *I* > 2σ(*I*)
graphite	*R*_int_ = 0.035
Detector resolution: 10.0 pixels mm^-1^	θ_max_ = 29.8°, θ_min_ = 4.4°
ω scans	*h* = −11→12
Absorption correction: multi-scan (*CrysAlis RED*; Oxford Diffraction, 2006)	*k* = −14→14
*T*_min_ = 0.748, *T*_max_ = 0.824	*l* = −18→19
11846 measured reflections	

### Refinement

**Table:**

Refinement on *F*^2^	Primary atom site location: structure-invariant direct methods
Least-squares matrix: full	Secondary atom site location: difference Fourier map
*R*[*F*^2^ > 2σ(*F*^2^)] = 0.077	Hydrogen site location: inferred from neighbouring sites
*wR*(*F*^2^) = 0.253	H atoms treated by a mixture of independent and constrained refinement
*S* = 1.05	*w* = 1/[σ^2^(*F*_o_^2^) + (0.1644*P*)^2^] where *P* = (*F*_o_^2^ + 2*F*_c_^2^)/3
6368 reflections	(Δ/σ)_max_ < 0.001
285 parameters	Δρ_max_ = 1.15 e Å^−^^3^
655 restraints	Δρ_min_ = −0.77 e Å^−^^3^

### Special details

**Table:**

Geometry. All e.s.d.'s (except the e.s.d. in the dihedral angle between two l.s. planes) are estimated using the full covariance matrix. The cell e.s.d.'s are taken into account individually in the estimation of e.s.d.'s in distances, angles and torsion angles; correlations between e.s.d.'s in cell parameters are only used when they are defined by crystal symmetry. An approximate (isotropic) treatment of cell e.s.d.'s is used for estimating e.s.d.'s involving l.s. planes.
Refinement. Refinement of *F*^2^ against ALL reflections. The weighted *R*-factor *wR* and goodness of fit *S* are based on *F*^2^, conventional *R*-factors *R* are based on *F*, with *F* set to zero for negative *F*^2^. The threshold expression of *F*^2^ > σ(*F*^2^) is used only for calculating *R*-factors(gt) *etc*. and is not relevant to the choice of reflections for refinement. *R*-factors based on *F*^2^ are statistically about twice as large as those based on *F*, and *R*- factors based on ALL data will be even larger.

### Fractional atomic coordinates and isotropic or equivalent isotropic displacement parameters (Å^2^)

**Table:**

	*x*	*y*	*z*	*U*_iso_*/*U*_eq_	Occ. (<1)
Zn1	0.05562 (7)	0.12433 (7)	0.50054 (5)	0.0340 (3)	
C1	−0.1270 (7)	−0.0328 (6)	0.6667 (4)	0.0348 (12)	
C2	−0.0490 (7)	−0.0070 (7)	0.7384 (5)	0.0393 (13)	
C3	−0.0831 (8)	−0.0639 (8)	0.8383 (5)	0.0466 (15)	
H3	−0.0290	−0.0493	0.8858	0.056*	
C4	0.0649 (7)	0.0818 (7)	0.7131 (5)	0.0414 (13)	
H4A	0.0126	0.1736	0.6901	0.050*	
H4B	0.1207	0.0693	0.7724	0.050*	
C5	0.3043 (7)	0.1208 (6)	0.6217 (5)	0.0378 (12)	
C6	0.3920 (8)	0.1117 (7)	0.7007 (6)	0.0465 (14)	
H6	0.3657	0.0661	0.7646	0.056*	
C7	0.5162 (8)	0.1685 (8)	0.6863 (6)	0.0509 (15)	
H7	0.5743	0.1609	0.7401	0.061*	
C8	0.5554 (8)	0.2371 (8)	0.5923 (6)	0.0489 (15)	
H8	0.6404	0.2755	0.5821	0.059*	
C9	0.4671 (7)	0.2484 (7)	0.5129 (6)	0.0454 (14)	
H9	0.4931	0.2953	0.4493	0.055*	
C10	0.3420 (7)	0.1915 (6)	0.5269 (5)	0.0375 (12)	
C11	0.3368 (7)	0.1223 (7)	0.3804 (5)	0.0397 (13)	
H11A	0.3798	0.0336	0.4223	0.048*	
H11B	0.4192	0.1649	0.3484	0.048*	
C12	0.2376 (7)	0.1125 (7)	0.3023 (5)	0.0397 (13)	
C13	0.2679 (8)	0.1641 (8)	0.2016 (5)	0.0513 (15)	
H13	0.3412	0.2163	0.1820	0.062*	
C14	0.1915 (9)	0.1397 (8)	0.1302 (5)	0.0530 (16)	
C15	0.2210 (10)	0.2038 (10)	0.0195 (6)	0.070 (2)	
C16	0.0903 (18)	0.274 (2)	−0.0392 (19)	0.106 (6)*	0.448 (12)
H16A	0.0088	0.2276	−0.0169	0.159*	0.448 (12)
H16B	0.1117	0.2812	−0.1072	0.159*	0.448 (12)
H16C	0.0625	0.3617	−0.0331	0.159*	0.448 (12)
C17	0.3466 (18)	0.269 (2)	−0.0069 (17)	0.078 (5)*	0.448 (12)
H17A	0.4286	0.2207	0.0380	0.117*	0.448 (12)
H17B	0.3183	0.3576	−0.0035	0.117*	0.448 (12)
H17C	0.3774	0.2721	−0.0730	0.117*	0.448 (12)
C18	0.276 (3)	0.084 (2)	−0.022 (2)	0.101 (6)*	0.448 (12)
H18A	0.3649	0.0287	0.0136	0.151*	0.448 (12)
H18B	0.2996	0.1178	−0.0908	0.151*	0.448 (12)
H18C	0.1983	0.0336	−0.0136	0.151*	0.448 (12)
C16'	0.146 (3)	0.3448 (14)	−0.0045 (17)	0.106 (5)*	0.552 (12)
H16D	0.0426	0.3503	0.0157	0.158*	0.552 (12)
H16E	0.1510	0.3878	−0.0745	0.158*	0.552 (12)
H16F	0.1963	0.3879	0.0297	0.158*	0.552 (12)
C17'	0.373 (3)	0.163 (3)	0.0001 (18)	0.106 (5)*	0.552 (12)
H17D	0.3988	0.0676	0.0228	0.160*	0.552 (12)
H17E	0.4347	0.1978	0.0340	0.160*	0.552 (12)
H17F	0.3908	0.1950	−0.0697	0.160*	0.552 (12)
C18'	0.154 (2)	0.1537 (17)	−0.0493 (15)	0.083 (4)*	0.552 (12)
H18D	0.0463	0.1770	−0.0471	0.124*	0.552 (12)
H18E	0.1845	0.0589	−0.0303	0.124*	0.552 (12)
H18F	0.1882	0.1926	−0.1152	0.124*	0.552 (12)
C19	−0.1180 (8)	0.3676 (7)	0.4525 (7)	0.0524 (18)	
C20	−0.2183 (11)	0.4959 (9)	0.3983 (7)	0.074 (3)	
H20A	−0.2489	0.4915	0.3365	0.110*	
H20B	−0.1644	0.5672	0.3861	0.110*	
H20C	−0.3057	0.5112	0.4376	0.110*	
C21	0.192 (3)	0.573 (3)	0.260 (2)	0.107 (7)*	0.50
H21A	0.2097	0.6567	0.2627	0.129*	0.50
H21B	0.0950	0.5895	0.2286	0.129*	0.50
C22	0.308 (3)	0.525 (3)	0.197 (2)	0.117 (7)*	0.50
H22A	0.3097	0.5925	0.1341	0.176*	0.50
H22B	0.2863	0.4467	0.1867	0.176*	0.50
H22C	0.4052	0.5031	0.2281	0.176*	0.50
C23	0.690 (2)	0.287 (2)	0.1224 (14)	0.081 (5)	0.50
H23A	0.7180	0.3421	0.0591	0.121*	0.50
H23B	0.5824	0.3020	0.1279	0.121*	0.50
H23C	0.7284	0.1956	0.1289	0.121*	0.50
C24	0.7513 (19)	0.3194 (16)	0.1974 (12)	0.058 (4)	0.50
H24A	0.7141	0.4124	0.1901	0.070*	0.50
H24B	0.8602	0.3056	0.1912	0.070*	0.50
O1	−0.0909 (5)	0.2773 (5)	0.4110 (4)	0.0537 (13)	
O2	−0.0635 (7)	0.3485 (6)	0.5334 (5)	0.0669 (15)	
O3	−0.1018 (5)	0.0163 (4)	0.5695 (3)	0.0338 (9)	
O4	0.1846 (10)	0.4916 (7)	0.3531 (5)	0.0344 (18)	0.50
H4	0.1170	0.5264	0.3826	0.052*	0.50
O5	0.7114 (8)	0.2386 (7)	0.2940 (5)	0.0252 (15)	0.50
H5	0.7511	0.2567	0.3367	0.038*	0.50
H2N	0.205 (8)	−0.030 (3)	0.652 (5)	0.050 (6)*	
H1N	0.225 (12)	0.271 (6)	0.394 (5)	0.095 (4)*	
N2	0.1736 (6)	0.0556 (5)	0.6353 (4)	0.0366 (11)	
N1	0.2504 (6)	0.2002 (5)	0.4438 (4)	0.0342 (11)	

### Atomic displacement parameters (Å^2^)

**Table:**

	*U*^11^	*U*^22^	*U*^33^	*U*^12^	*U*^13^	*U*^23^
Zn1	0.0304 (4)	0.0382 (4)	0.0374 (5)	−0.0082 (3)	0.0014 (3)	−0.0165 (3)
C1	0.031 (3)	0.042 (3)	0.036 (3)	−0.006 (2)	0.003 (2)	−0.019 (2)
C2	0.035 (3)	0.049 (3)	0.041 (3)	−0.009 (2)	0.001 (2)	−0.024 (3)
C3	0.043 (3)	0.065 (4)	0.038 (3)	−0.013 (3)	0.000 (3)	−0.022 (3)
C4	0.040 (3)	0.052 (3)	0.040 (3)	−0.015 (2)	0.003 (2)	−0.022 (2)
C5	0.029 (2)	0.044 (3)	0.047 (3)	−0.009 (2)	−0.001 (2)	−0.023 (2)
C6	0.040 (3)	0.058 (3)	0.049 (3)	−0.012 (3)	−0.002 (3)	−0.026 (3)
C7	0.038 (3)	0.063 (4)	0.061 (4)	−0.011 (3)	−0.009 (3)	−0.030 (3)
C8	0.032 (3)	0.061 (4)	0.060 (4)	−0.016 (3)	−0.005 (3)	−0.024 (3)
C9	0.036 (3)	0.054 (3)	0.053 (3)	−0.015 (3)	0.003 (3)	−0.023 (3)
C10	0.030 (2)	0.041 (3)	0.048 (3)	−0.008 (2)	0.000 (2)	−0.023 (2)
C11	0.032 (3)	0.052 (3)	0.044 (3)	−0.014 (2)	0.006 (2)	−0.024 (2)
C12	0.035 (3)	0.051 (3)	0.040 (3)	−0.015 (2)	0.007 (2)	−0.022 (3)
C13	0.048 (3)	0.067 (4)	0.046 (3)	−0.025 (3)	0.010 (3)	−0.021 (3)
C14	0.055 (3)	0.069 (4)	0.042 (3)	−0.021 (3)	0.009 (3)	−0.024 (3)
C15	0.071 (4)	0.095 (5)	0.050 (4)	−0.035 (4)	0.011 (3)	−0.021 (4)
C19	0.035 (4)	0.041 (4)	0.077 (5)	−0.008 (3)	0.003 (4)	−0.014 (4)
C20	0.077 (6)	0.055 (5)	0.083 (6)	0.014 (4)	−0.017 (5)	−0.027 (5)
C23	0.067 (10)	0.097 (12)	0.058 (9)	−0.011 (9)	0.004 (8)	0.000 (9)
C24	0.060 (9)	0.051 (8)	0.062 (9)	−0.008 (7)	−0.013 (7)	−0.016 (7)
O1	0.042 (3)	0.040 (3)	0.078 (4)	−0.001 (2)	−0.005 (2)	−0.021 (2)
O2	0.067 (4)	0.057 (3)	0.069 (4)	−0.002 (3)	−0.012 (3)	−0.014 (3)
O3	0.033 (2)	0.039 (2)	0.033 (2)	−0.0101 (17)	0.0037 (17)	−0.0157 (18)
O4	0.056 (5)	0.015 (3)	0.024 (4)	−0.001 (3)	0.009 (4)	0.000 (3)
O5	0.026 (4)	0.024 (4)	0.027 (4)	−0.008 (3)	−0.004 (3)	−0.008 (3)
N2	0.034 (3)	0.042 (3)	0.040 (3)	−0.010 (2)	0.000 (2)	−0.019 (2)
N1	0.032 (3)	0.037 (3)	0.037 (3)	−0.005 (2)	0.000 (2)	−0.017 (2)

### Geometric parameters (Å, °)

**Table:**

Zn1—O1	2.025 (5)	C16—H16A	0.9600
Zn1—O3	2.033 (4)	C16—H16B	0.9600
Zn1—O3^i^	2.043 (4)	C16—H16C	0.9600
Zn1—N2	2.100 (5)	C17—H17A	0.9600
Zn1—N1	2.104 (5)	C17—H17B	0.9600
Zn1—Zn1^i^	3.0670 (13)	C17—H17C	0.9600
C1—O3	1.341 (7)	C18—H18A	0.9600
C1—C2	1.407 (8)	C18—H18B	0.9600
C1—C12^i^	1.412 (9)	C18—H18C	0.9600
C2—C3	1.403 (9)	C16'—H16D	0.9600
C2—C4	1.501 (9)	C16'—H16E	0.9600
C3—C14^i^	1.370 (10)	C16'—H16F	0.9600
C3—H3	0.9300	C17'—H17D	0.9600
C4—N2	1.496 (8)	C17'—H17E	0.9600
C4—H4A	0.9700	C17'—H17F	0.9600
C4—H4B	0.9700	C18'—H18D	0.9600
C5—C6	1.385 (9)	C18'—H18E	0.9600
C5—C10	1.386 (9)	C18'—H18F	0.9600
C5—N2	1.470 (8)	C19—O2	1.223 (10)
C6—C7	1.363 (10)	C19—O1	1.276 (9)
C6—H6	0.9300	C19—C20	1.497 (11)
C7—C8	1.374 (11)	C20—H20A	0.9600
C7—H7	0.9300	C20—H20B	0.9600
C8—C9	1.386 (10)	C20—H20C	0.9600
C8—H8	0.9300	C21—O4	1.34 (3)
C9—C10	1.371 (9)	C21—C22	1.47 (3)
C9—H9	0.9300	C21—H21A	0.9700
C10—N1	1.456 (8)	C21—H21B	0.9700
C11—N1	1.511 (7)	C22—H22A	0.9600
C11—C12	1.517 (9)	C22—H22B	0.9600
C11—H11A	0.9700	C22—H22C	0.9600
C11—H11B	0.9700	C23—C24	1.40 (2)
C12—C13	1.398 (10)	C23—H23A	0.9600
C12—C1^i^	1.412 (9)	C23—H23B	0.9600
C13—C14	1.387 (10)	C23—H23C	0.9600
C13—H13	0.9300	C24—O5	1.446 (18)
C14—C3^i^	1.370 (10)	C24—H24A	0.9700
C14—C15	1.540 (11)	C24—H24B	0.9700
C15—C17'	1.40 (2)	O3—Zn1^i^	2.043 (4)
C15—C16	1.426 (11)	O4—H4	0.8200
C15—C17	1.426 (11)	O5—H5	0.8200
C15—C18'	1.475 (17)	N2—H2N	0.87 (3)
C15—C16'	1.479 (11)	N1—H1N	0.86 (3)
C15—C18	1.57 (2)		
			
O1—Zn1—O3	96.05 (19)	H16A—C16—H16B	109.5
O1—Zn1—O3^i^	105.62 (19)	C15—C16—H16C	109.5
O3—Zn1—O3^i^	82.40 (17)	H16A—C16—H16C	109.5
O1—Zn1—N2	144.5 (2)	H16B—C16—H16C	109.5
O3—Zn1—N2	88.07 (18)	C15—C17—H17A	109.5
O3^i^—Zn1—N2	109.82 (19)	C15—C17—H17B	109.5
O1—Zn1—N1	95.6 (2)	H17A—C17—H17B	109.5
O3—Zn1—N1	168.18 (18)	C15—C17—H17C	109.5
O3^i^—Zn1—N1	92.33 (17)	H17A—C17—H17C	109.5
N2—Zn1—N1	83.8 (2)	H17B—C17—H17C	109.5
O1—Zn1—Zn1^i^	104.44 (14)	C15—C18—H18A	109.5
O3—Zn1—Zn1^i^	41.32 (11)	C15—C18—H18B	109.5
O3^i^—Zn1—Zn1^i^	41.08 (11)	H18A—C18—H18B	109.5
N2—Zn1—Zn1^i^	101.75 (15)	C15—C18—H18C	109.5
N1—Zn1—Zn1^i^	132.53 (13)	H18A—C18—H18C	109.5
O3—C1—C2	122.6 (6)	H18B—C18—H18C	109.5
O3—C1—C12^i^	118.4 (5)	C15—C16'—H16D	109.5
C2—C1—C12^i^	119.0 (6)	C15—C16'—H16E	109.5
C3—C2—C1	118.2 (6)	H16D—C16'—H16E	109.5
C3—C2—C4	118.5 (6)	C15—C16'—H16F	109.5
C1—C2—C4	123.2 (6)	H16D—C16'—H16F	109.5
C14^i^—C3—C2	123.6 (6)	H16E—C16'—H16F	109.5
C14^i^—C3—H3	118.2	C15—C17'—H17D	109.5
C2—C3—H3	118.2	C15—C17'—H17E	109.5
N2—C4—C2	112.9 (5)	H17D—C17'—H17E	109.5
N2—C4—H4A	109.0	C15—C17'—H17F	109.5
C2—C4—H4A	109.0	H17D—C17'—H17F	109.5
N2—C4—H4B	109.0	H17E—C17'—H17F	109.5
C2—C4—H4B	109.0	C15—C18'—H18D	109.5
H4A—C4—H4B	107.8	C15—C18'—H18E	109.5
C6—C5—C10	119.2 (6)	H18D—C18'—H18E	109.5
C6—C5—N2	121.9 (6)	C15—C18'—H18F	109.5
C10—C5—N2	118.9 (5)	H18D—C18'—H18F	109.5
C7—C6—C5	121.1 (7)	H18E—C18'—H18F	109.5
C7—C6—H6	119.5	O2—C19—O1	120.5 (7)
C5—C6—H6	119.5	O2—C19—C20	122.1 (7)
C6—C7—C8	119.9 (7)	O1—C19—C20	117.4 (8)
C6—C7—H7	120.0	C19—C20—H20A	109.5
C8—C7—H7	120.0	C19—C20—H20B	109.5
C7—C8—C9	119.4 (6)	H20A—C20—H20B	109.5
C7—C8—H8	120.3	C19—C20—H20C	109.5
C9—C8—H8	120.3	H20A—C20—H20C	109.5
C10—C9—C8	121.0 (7)	H20B—C20—H20C	109.5
C10—C9—H9	119.5	O4—C21—C22	116 (2)
C8—C9—H9	119.5	O4—C21—H21A	108.2
C9—C10—C5	119.4 (6)	C22—C21—H21A	108.2
C9—C10—N1	121.4 (6)	O4—C21—H21B	108.2
C5—C10—N1	119.2 (5)	C22—C21—H21B	108.2
N1—C11—C12	112.1 (5)	H21A—C21—H21B	107.4
N1—C11—H11A	109.2	C21—C22—H22A	109.5
C12—C11—H11A	109.2	C21—C22—H22B	109.5
N1—C11—H11B	109.2	H22A—C22—H22B	109.5
C12—C11—H11B	109.2	C21—C22—H22C	109.5
H11A—C11—H11B	107.9	H22A—C22—H22C	109.5
C13—C12—C1^i^	119.9 (6)	H22B—C22—H22C	109.5
C13—C12—C11	121.2 (6)	C24—C23—H23A	109.5
C1^i^—C12—C11	118.3 (6)	C24—C23—H23B	109.5
C14—C13—C12	121.6 (7)	H23A—C23—H23B	109.5
C14—C13—H13	119.2	C24—C23—H23C	109.5
C12—C13—H13	119.2	H23A—C23—H23C	109.5
C3^i^—C14—C13	117.6 (7)	H23B—C23—H23C	109.5
C3^i^—C14—C15	121.7 (7)	C23—C24—O5	111.0 (14)
C13—C14—C15	120.6 (7)	C23—C24—H24A	109.4
C17'—C15—C16	135.3 (16)	O5—C24—H24A	109.4
C17'—C15—C17	45.7 (11)	C23—C24—H24B	109.4
C16—C15—C17	113.1 (11)	O5—C24—H24B	109.4
C17'—C15—C18'	98.4 (13)	H24A—C24—H24B	108.0
C16—C15—C18'	56.2 (11)	C19—O1—Zn1	106.4 (5)
C17—C15—C18'	122.3 (14)	C1—O3—Zn1	128.5 (4)
C17'—C15—C16'	123.3 (15)	C1—O3—Zn1^i^	113.8 (4)
C16—C15—C16'	50.1 (11)	Zn1—O3—Zn1^i^	97.60 (17)
C17—C15—C16'	78.5 (13)	C21—O4—H4	109.5
C18'—C15—C16'	105.0 (10)	C24—O5—H5	109.5
C17'—C15—C14	108.8 (12)	C5—N2—C4	114.9 (5)
C16—C15—C14	115.4 (13)	C5—N2—Zn1	107.9 (4)
C17—C15—C14	118.0 (11)	C4—N2—Zn1	107.6 (4)
C18'—C15—C14	116.1 (10)	C5—N2—H2N	109 (5)
C16'—C15—C14	105.7 (12)	C4—N2—H2N	107 (5)
C17'—C15—C18	58.0 (8)	Zn1—N2—H2N	110 (5)
C16—C15—C18	102.3 (11)	C10—N1—C11	110.9 (5)
C17—C15—C18	99.6 (14)	C10—N1—Zn1	108.1 (4)
C18'—C15—C18	46.8 (10)	C11—N1—Zn1	110.1 (3)
C16'—C15—C18	145.6 (14)	C10—N1—H1N	124 (7)
C14—C15—C18	105.2 (12)	C11—N1—H1N	93 (7)
C15—C16—H16A	109.5	Zn1—N1—H1N	109 (7)
C15—C16—H16B	109.5		

Symmetry codes: (i) −*x*, −*y*, −*z*+1.

### Hydrogen-bond geometry (Å, °)

**Table:**

*D*—H···*A*	*D*—H	H···*A*	*D*···*A*	*D*—H···*A*
C4—H4A···O2	0.97	2.45	3.246 (10)	139
N1—H1N···O4	0.86 (7)	2.23 (7)	2.952 (9)	141 (6)
N2—H2N···O5^ii^	0.87 (4)	2.13 (4)	2.999 (9)	175 (4)
O4—H4···O2^iii^	0.82	2.06	2.768 (10)	145
O5—H5···O1^iv^	0.82	1.92	2.700 (9)	159

Symmetry codes: (ii) −*x*+1, −*y*, −*z*+1; (iii) −*x*, −*y*+1, −*z*+1; (iv) *x*+1, *y*, *z*.

## References

[bb1] Bazzicalupi, C., Bencini, A., Bianchi, A., Fusi, V., Giorgi, C., Paoletti, P., Valtancoli, B. & Zanchi, D. (1997). *Inorg. Chem.* **36**, 2784–2790. 10.1021/ic961521j 11669912

[bb2] Burley, S. K., David, P. R., Taylor, A. & Lipscomb, W. N. (1990). *Proc. Natl Acad. Sci. USA*, **87**, 6878–6882. 10.1073/pnas.87.17.6878 PMC54641 2395881

[bb3] Dutta, B., Bag, P., Flörke, U. & Nag, K. (2005). *Inorg. Chem* **44**, 147–157. 10.1021/ic049056a 15627370

[bb4] Lipscomb, W. N. & Straeter, N. (1996). *Chem. Rev.* **96**, 2375–2434. 10.1021/cr950042j 11848831

[bb5] Liu, J., Ma, J.-F., Li, S.-L. & Ping, G.-J. (2007). *Acta Cryst.* E**63**, m1954.

[bb6] Oxford Diffraction (2006). *CrysAlis CCD* and *CrysAlis RED* Oxford Diffraction Ltd, Abingdon, England.

[bb7] Roderick, S. & Mathews, B. W. (1993). *Biochemistry*, **32**, 3907–3912. 10.1021/bi00066a009 8471602

[bb8] Sheldrick, G. M. (2008). *Acta Cryst.* A**64**, 112–122. 10.1107/S0108767307043930 18156677

[bb9] Tian, Y. Q., Tong, J., Frenzen, G. & Sun, J. Y. (1999). *J. Org. Chem.* **64**, 1442–1446. 10.1021/jo9809736 11674201

